# The British Hospitals Association

**Published:** 1909-10-16

**Authors:** 


### THE BRITISH HOSPITALS ASSOCIATION.

### THE HOSPITAL CONGRESS ADOPTED.

The Hospitals Association was founded in the year 1884 for the promotion of co-operation among those connected with, and interested in, hospitals and kindred charities, to provide facilities for the discussion of matters connected with hospital management, and for the collection and diffusion of information of interest to those associated with institutions and their work.

### What the Association has Achieved.

The association was managed by a council of thirty-one members under numerous presidents, the present president being Mr. Thomas Bryant, F.R.C.S., while the Chairman of Council is the Duke of Leeds. For upwards of twenty years it has done excellent work and proved its usefulness in various ways. It has been associated with the inception of the Royal National Pension Fund for Nurses, has organised an efficient street ambulance service for London which has been in active work for many years under the able management of Mr. Thomas Ryan, and supported through the munificence of the late Mr. Bischoffsheim. This ambulance work, it is satisfactory to note, has crystallised this year, through Sir William Collins's able management, in the passing of a special Act of Parliament giving authority to the London County Council to take over and work an ambulance service for London, which result may fairly be placed in good measure to the work done through the instrumentality of the association. Further, the association has secured the passing of two important Acts, chief among which may be mentioned the Act to amend the Mortmain Act which removed certain restrictions upon charitable gifts, and the second which removed an injustice to the nurses engaged in Poor-Law service by permitting them to contract themselves out. At the various meetings of the association papers of great merit and on subjects of the highest importance in hospital politics have been read and discussed, and there can be no doubt that while the association was actively at work it served public purposes so well that those who are acquainted with what it has done must regret that it has remained *in statu quo* during recent years. New associations doing somewhat similar, though not identical, work have arisen. Of these perhaps the Central Hospital Council and the Hospital Officers' Association are the most important. The former was founded in 1897, and consists of representatives from the chief London hospitals having medical schools attached to them, and its object is to consider matters in which the hospitals may have a common interest. The Hospital Officers' Society, an important and useful organisation, is mainly of interest to the junior officers of our hospitals.

### The Need for Future Work.

We have always felt that the exact field of work covered by the Hospitals Association has not been exploited by any of the newer ones. We fully recognise the utility of these newer organisations, but while admitting their usefulness, we are at the same time conscious of the fact that there is still need for an association that will embrace the managers of all the voluntary hospitals in the British Islands, and that will carry on the policy of the old association and work in the best interests of the voluntary hospitals. In America the American Hospitals Association is doing excellent work, and has proved of immense service and benefit to hospital administrators in the United States and Canada, while the need for an association on somewhat similar, if not indeed identical, lines has never been so greatly felt in England as at the present moment.

### The British Hospitals Association.

For these reasons it gives us the most cordial gratification to be able to announce that the movement to reorganise the Hospitals Association, which was set on foot by some of the old members, has been most successful. Dr. D. J. Mackintosh, of the Western Infirmary, Glasgow, and Mr. E. W. Morris, of the London Hospital, Whitechapel, E., who have undertaken the onerous duties of secretaries to the reorganised association, have circularised all the old members and all the managers of the various British hospitals, and the response to their appeal has been gratifyingly prompt and, in 99 per cent. of cases, wholeheartedly sympathetic. Through the courtesy of the joint secretaries we are enabled to give a summary of the scope and objects of the new association. It is proposed to call it "The British Hospitals Association," so as to include in it the managers of all the voluntary hospitals in England, Wales, Scotland, and Ireland. But the scope of the Association is to be somewhat more extended. It will embrace the work of the various infirmaries, Poor Law boards, guardians, parish councils, and dispensaries, and efforts will be made to have these various bodies represented on the council. Membership, at least at first, will be restricted to the chairmen of hospitals and dispensary boards and committees, to superintendents, secretaries and assistant-secretaries, and to matrons and assistant matrons.

### Problems that Await Discussion.

The Association will arrange meetings from time to time at different centres for the discussion of subjects of interest to hospital managers. There are many such subjects, and to show how wide the field is and how useful the discussions may be, one has only to glance at the agenda and at the reports of the last sessional meeting of the American Association. Subjects for discussion suggest themselves readily enough, even when the limitation is made that all matters discussed must be of general and paramount importance. Trivialities need not absorb the attention of such an annual or a biennial congress, but the following preliminary list of possible subjects for discussion will give an idea of the problems which may usefully demand attention.

1. The report of the Poor Law Commission and its relation to voluntary hospitals.
2. The voluntary system, its advantages and its disadvantages.
3. Is it possible to form any standard of hospital efficiency?
4. Hospital almoners and their work.
5. The hospital treatment of school children.
6. The municipalisation of hospitals.
7. The relations between hospitals and their medical schools.
8. The training of hospital nurses.
9. The kind and extent of control which may be exercised by a lay committee over the medical staff.
10. Abuse of hospitals (*a*) by the general practitioner, and (*b*) by the public.
11. Payment of the staff.
12. Workmen's compensation. Hospitals and insurance companies.
13. Hospitals as provident dispensaries.
14. The establishment of central hospital buying and distributing departments.
15. International hospital correspondence.
16. Unification of charities.
17. Training of hospital administrators.

We need not elaborate each of these points in detail: their importance is evident to everyone who has had any experience of hospital work. Take one instance only, the establishment of a central buying station for hospitals. Mr. Morris, in discussing the point, draws attention to the fact that every year the several dispensers or chemists of the large London hospitals devote a portion of their time to the careful analysis of drug samples. These samples are very often sent in by the same firms, from the same stocks, and there is in consequence a great waste of time, energy, and trouble on the part of the chemists attached to the hospitals. All this waste might be, and could be, prevented by the establishment of a central buying station where the various samples could be analysed on behalf of all the hospitals. The whole question will bear discussion, and from such a discussion much good might result. And this, it will be observed, is only one of the many subjects which demand attention.

### The Library.

It is proposed to keep up the already existing central library and reference bureau, which at present contains a collection of hospital literature which is practically unique in England, and which will remain available for reference. A Central Council will be established on which will sit representatives of provincial institutions.

This, briefly, is an outline of the reorganised British Hospitals Association. All particulars regarding its work and objects may be obtained on application to the Honorary Secretaries at the offices of the Association, 28 Southampton Street, Strand, London, W.C., who will be glad to receive the support of all interested in hospital management. Although no definite date has been fixed for the first meeting of the new association, it is expected that a preliminary meeting will take place at an early date, and that the final programme of the association will then be arranged, the date for the first general meeting fixed, and other important essentials determined.

